# Supplementary material for: Unveiling multifunctional synthetic boundaries for enhanced mechanical and electrochemical performance in densified thick composite electrodes
Source: Nat Commun. 2025 Oct 29;16:9489. doi: 10.1038/s41467-025-65257-2 (PMC12568943; doi:10.1038/s41467-025-65257-2)
Supplement: Supplementary file 1 — Supplementary Information [file 41467_2025_65257_MOESM1_ESM.pdf]

Supplementary Information

Unveiling Multifunctional Synthetic Boundaries for Enhanced Mechanical and Electrochemical Performance in Densified Thick Composite Electrodes

This file includes:

Supplementary Text
Supplementary Figs. 1-28
Supplementary Tables 1-10

Supplementary Text

TGA analysis

Thermal analysis in airflow conditions revealed the sequential decomposition behavior of the composite components. The thermal decomposition of the PILG phase occurs in a complex and overlapping manner under oxidative conditions, we performed derivative thermogravimetric (DTG) analysis and multi-step deconvolution to quantitatively separate each contribution [1,2]. Based on this approach, we calculated the individual weight ratios of the PILG components, as summarized in Supplementary Figs. 5, 11 and 12, and Supplementary Tables 2, 3. Notably, the decomposition temperature of carbon additives slightly elevated due to surface functionalization by the PILG matrix, further confirming strong interfacial interactions [3-5].

Estimation of the porosity of thick electrodes

The density of NMC811 (ρ_{NMC}) is 4.87 g cm^{-3}, and the density of the carbon components, including graphene and CNF (ρ_{carbon}), is assumed to be 2.0 g cm^{-3}. The density of the PILG component is assumed to be 1.6 g cm^{-3}. The specific weight fractions of components were determined by TGA results and analysis (see Supplementary Fig. 11, and Supplementary Tables 2, 3). The theoretical density of the composite is calculated as follows:

$$\rho_{cal.} = \frac{m_{composite}}{\frac{m_{NMC}}{\rho_{NMC}} + \frac{m_{carbons}}{\rho_{carbons}} + \frac{m_{PILG}}{\rho_{PILG}}} = 3.60 \text{ g cm}^{-3} \qquad (1)$$

The measured density of the densified composites ($\rho_{exp.}$) is 3.0 g cm^{-3} for NMC811-PILG thick electrodes, obtained by determining the geometrical parameters and electrode mass. Hence, the relative density of the NMC811-PILG electrode is 87.6%, and the corresponding porosity is 12.4%.

Electrochemical Characterizations

Galvanostatic intermittent titration technique (GITT) test. To determine the Li^+ diffusion kinetics for various electrodes, the assembled cells were subjected to a GITT pulse current at 0.1 C rate for 30 minutes (t). After each titration step, the cells were allowed to relax for 4 hours. This procedure was repeated until the cut-off potentials (2.7 V and 4.2 V) were reached.

The diffusion coefficient was calculated using the following equation:

$$\widetilde{D}_i = \frac{4}{\pi}\left(\frac{I_0 V_m}{FS}\right)^2\left(\frac{dE/dx}{dE/dt^{1/2}}\right)^2,\ t \ll \frac{L^2}{\widetilde{D}_i} \quad (3)$$

where $\widetilde{D}_i$ (cm^2 s^{-1}) is the chemical diffusion coefficient of the studied species i, V_m(cm^3 mol^{-1}) is the molar volume of the active material. F is the Faraday constant, I_0 is the applied current, S (cm^2) is the contact area between the electrode and the electrolyte, and L (cm) is the diffusion length. Given the porosity of the thick electrodes in this work is less than 15 %, the contact area S is assumed to be the geometric area of the densified pellet. The equation can be further simplified as:

$$\widetilde{D}_i = \frac{4}{\pi t}\left(\frac{n_m V_m}{S}\right)^2\left(\frac{E_4 - E_0}{E_2 - E_1}\right)^2 \quad (4)$$

where n_m (mol) is the number of moles. During a GITT pulse, there is a steep potential increase when switching on the current, known as the IR drop $E_1 - E_0$. When the current is switched off, the IR drop is $E_2 - E_3$. E_4 is the potential at the end of the following relaxation.

EIS symmetric cell characterization. The EIS symmetric cell characterization was conducted to determine the ionic resistance. Symmetric cells were assembled using two identical composite electrodes with thicknesses corresponding to practical mass loadings ranging from 40 to 100 mg cm^{-2}. Potentiostatic EIS measurements were performed across a frequency range of 1MHz to 0.01Hz to characterize the nonfaradaic process at a 0% state of charge (SOC).

According to the theory of the transmission line model (TLM), the ionic diffusion resistance in the electrode pores can be derived [6]. Theoretically, the derived Nyquist plots exhibit a 45 slope in the frequency region of approximately 5 to 100Hz and quasi-vertical lines at lower frequencies (<1Hz). This transition indicates constant resistance Z_ω in the low-frequency region. The limiting values of the real (Z'_ω) and imaginary (Z''_ω) parts as $\omega \rightarrow 0$ can be expressed by the Equations (5) and (6) [7]:

$$Z'_{\omega \rightarrow 0} = \frac{R_{ion}}{3} \quad (5)$$

$$Z''_{\omega \rightarrow 0} = \frac{1}{\omega C_{dl}} \quad (6)$$

where R_{ion} is a characteristic parameter describing lithium-ion diffusion in electrode pores. In the 45-degree region, the real part equals $R_{ion}/3$.

Cell-level energy density calculations

The areal energy density is calculated by integrating the voltage-capacity curve during the discharge process using the following equation:

$$Areal\ Energy\ Density = \int U \times C_{cell} \qquad (7)$$

where U is the operating cell voltage during discharge, and C_{cell} is the areal cell capacity. For the NMC811-PILG positive electrode with a thickness of 200 µm, the areal energy density is calculated as 34 mWh cm^{-2}

Specific energy and volumetric energy density are calculated based on the assumptions of the cell configuration, including only the cathode, anode, separator, electrolyte, and current collectors, as reported by Ju et al., [7]. **The specific energy** is calculated using the following equation:

$$Specific\ Energy = \frac{\int U \times C_{cell}}{m_{positive\ electrode} + m_{negative\ electrode} + m_{separator} + m_{electrolyte} + m_{current\ collector}}$$

(8)

where $m_{positive\ electrode}$, $m_{negative\ electrode}$, $m_{separator}$, $m_{electrolyte}$, and $m_{current\ collector}$ are the areal masses of the positive electrode, negative electrode, separator, electrolyte, and current collector, respectively. The electrolyte is assumed to accommodate the residual pores inside the cell components, with no extra volume required.

For the dense NMC811-PILG positive electrode with a thickness of 200 µm, the specific energy is calculated as:

$$E_{NMC811-PILG\ (200\ \mu m)} = \frac{34\ \text{mWh cm}^{-2}}{62.6\ \text{mg cm}^{-2} + 25.3\ \text{mg cm}^{-2} + 1.2\ \text{mg cm}^{-2} + 1.1 \times (1.0 + 0.007)\ \text{mg cm}^{-2} + 4.2\ \text{mg cm}^{-2}} = 362\ \text{Wh kg}^{-1}$$

In this calculation, $m_{negative\ electrode}$ is 25.3 mg cm^{-2} (lithium metal chip with a thickness of 0.2 mm), $m_{separator}$ is 1.2 mg cm^{-2} (20 µm thick monolayer PE separator with a porosity of 0.4), $m_{electrolyte}$ is $1.1 \times (1.0 + V_{P,positive\ electrode})$ mg cm^{-2}, $V_{P,positive\ electrode}$ iis the pore volume

of the positive electrode (mm^3), and $m_{current\ collector}$ is $4.2\ \mathrm{mg\ cm^{-2}}$ (16 μm thick aluminum foil).

The volumetric energy density is calculated using the following equation:

$$Vol.\,Energy\ Density = \frac{\int U \times C_{cell}}{V_{positive\ electrode} + V_{negative\ electrode} + V_{separator} + V_{electrolyte} + V_{current\ collector}}$$

(9)

where $V_{positive\ electrode}$, $V_{negative\ electrode}$, $V_{separator}$, $V_{electrolyte}$, and $V_{current\ collector}$ are the volume of the positive electrode, negative electrode, separator, electrolyte, and current collector, respectively.

For the dense NMC811-PILG positive electrode with a thickness of 200 μm, the volumetric energy density is calculated as:

$$E_{vol.,NMC811-PILG\ (200\ \mu m)} = \frac{34\ \mathrm{mWh\ cm^{-2}}}{0.2\ \mathrm{mm} + 0.25\ \mathrm{mm} + 0.02\ \mathrm{mm} + 0.016\ \mathrm{mm}} = 704\ \mathrm{Wh\ L^{-1}}$$

Here, the thickness of the electrolyte is assumed to be integrated with the separator and electrodes, with no extra volume expansion.

Energy consumption

The energy consumption for evaluating the economy of this densification process was calculated by considering the energy input, which includes mechanical uniaxial pressing and thermal heating. The mechanical energy input was estimated using the equation $W = Fd$, where F is the force, and d is the displacement. The force F is related to the applied pressure (400 MPa), and d (~0.5 mm) is the thickness difference before and after the densification process. The thermal energy input was monitored via the voltage and current during the process. Energy consumption for spark plasma sintering (SPS) and conventional sintering processes was based on the furnace heating profile reported by Heidary et al [8], assuming the furnace system had ideal insulation.

Supplementary Figures

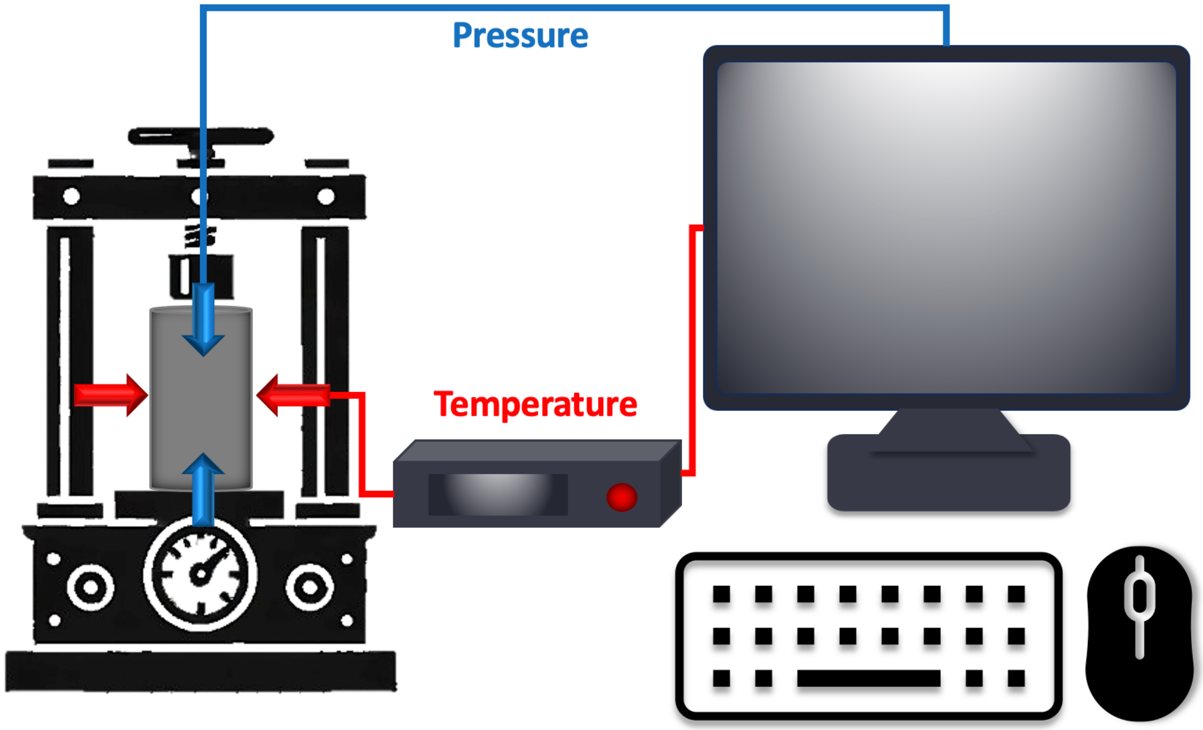

Supplementary Fig. 1. The experimental configuration of geology-inspired densification process.

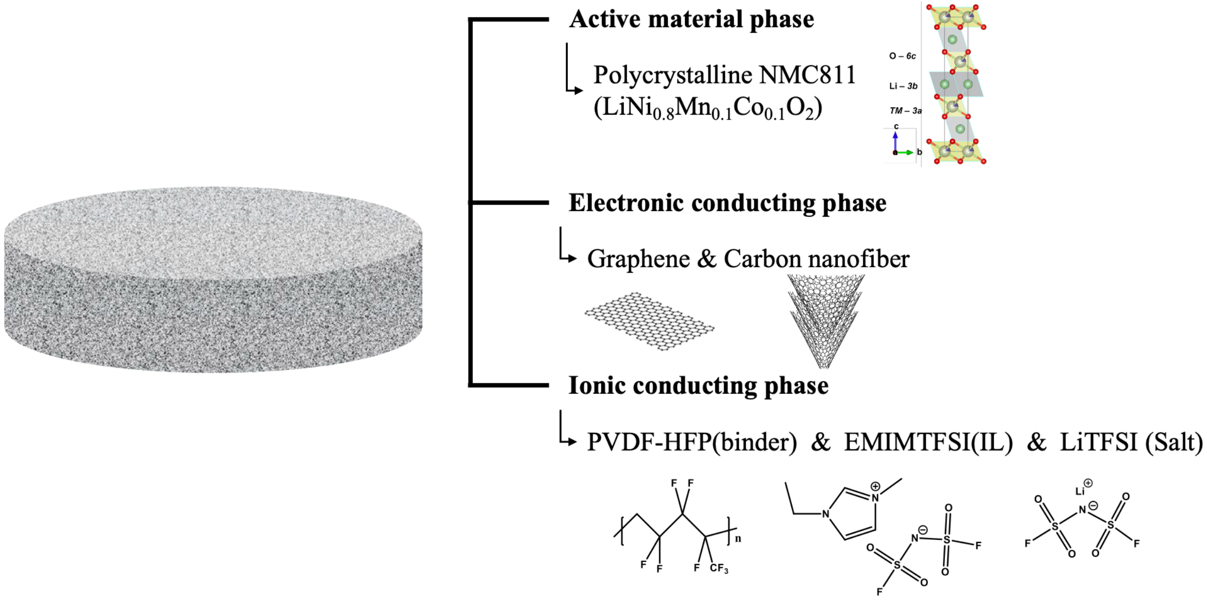

Supplementary Fig. 2. Schematic illustration of the chemical composition of each phase in the highly densified NMC811-PILG composite positive electrode.

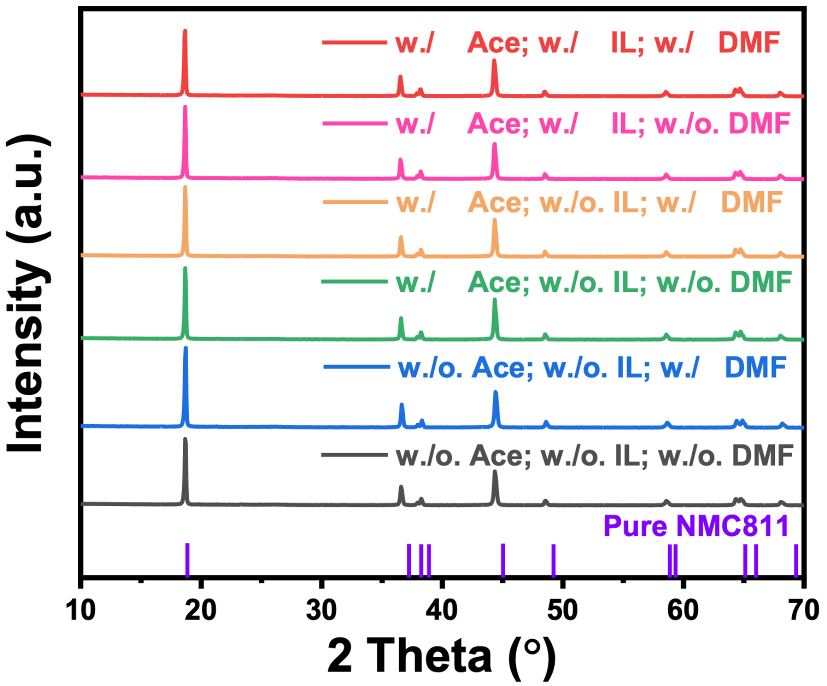

Supplementary Fig. 3. The X-ray diffraction (XRD) results of composite pellets densified using various liquids, including acetone, DMF, and/or ionic liquid (IL).

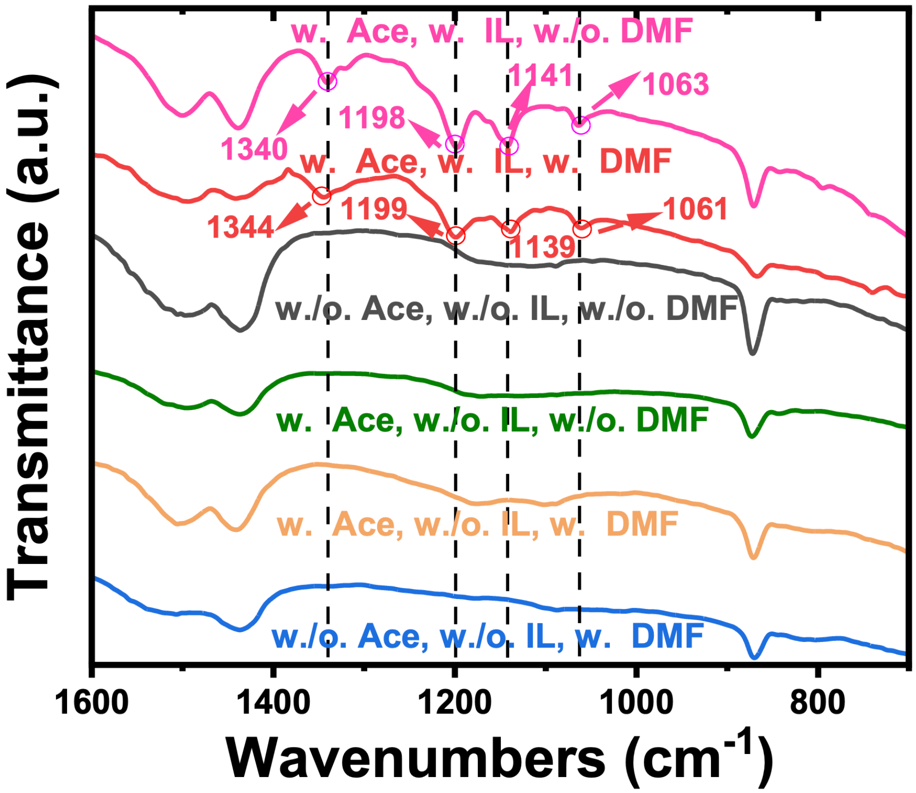

Supplementary Fig. 4. The Fourier Transform Infrared Spectroscopy (FT-IR) result of composite pellets densified using various liquids, including acetone, DMF, and/or ionic liquid (IL).

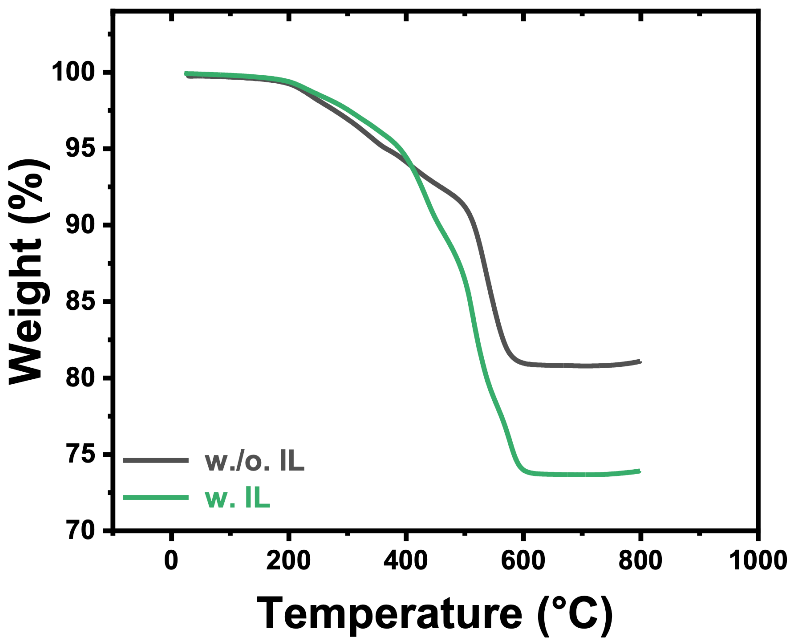

Supplementary Fig. 5. The TGA curves of the densified composite pellets with and without ionic liquid (IL).

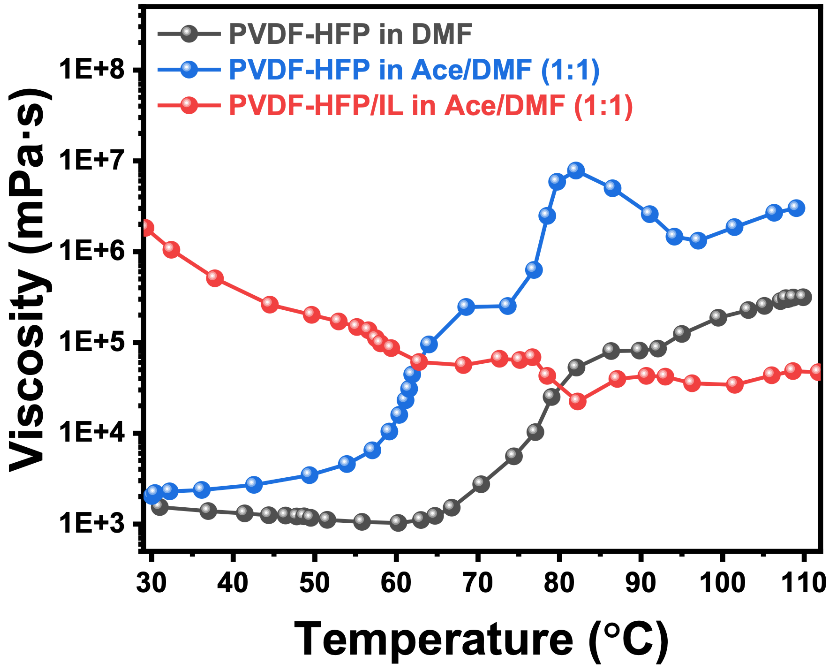

Supplementary Fig. 6. The viscosity measurement of the PVDF-HFP copolymer solution (20 wt%) with different solvents and with or without IL addition.

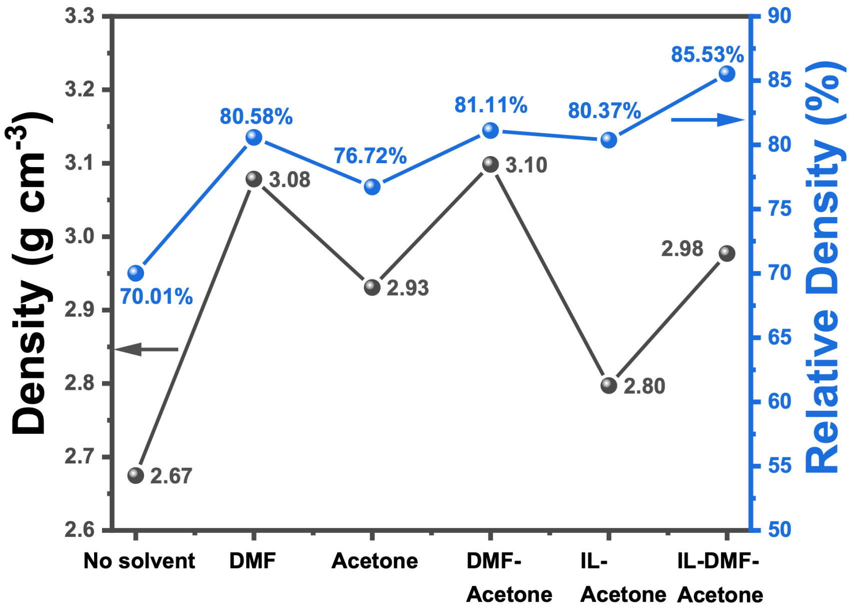

Supplementary Fig. 7. The measured density and calculated relative density for the densified samples under different conditions. The densification process conducted without acetone, DMF, and IL is referred to as hot pressing.

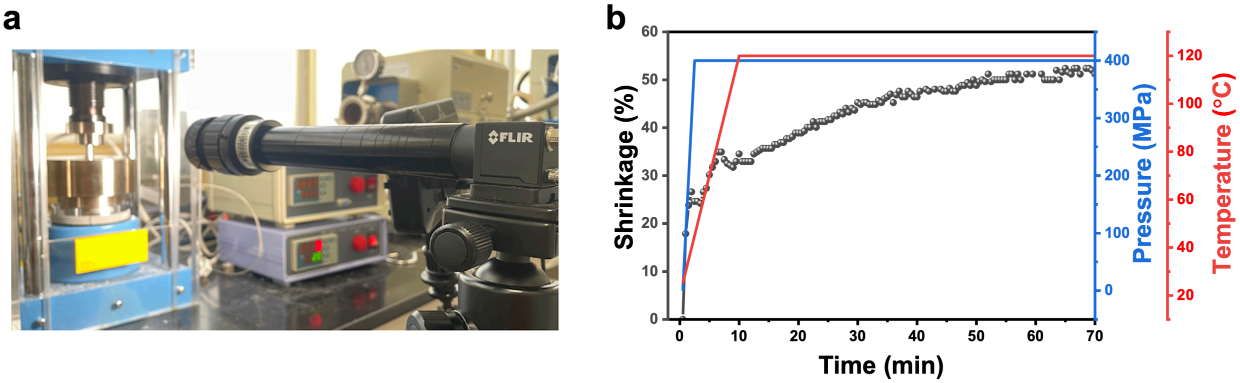

Supplementary Fig. 8. Monitoring of densification process. a, The setup for monitoring of densification process. **b,** The profiles of temperature, pressure and linear shrinkage during the solidification process.

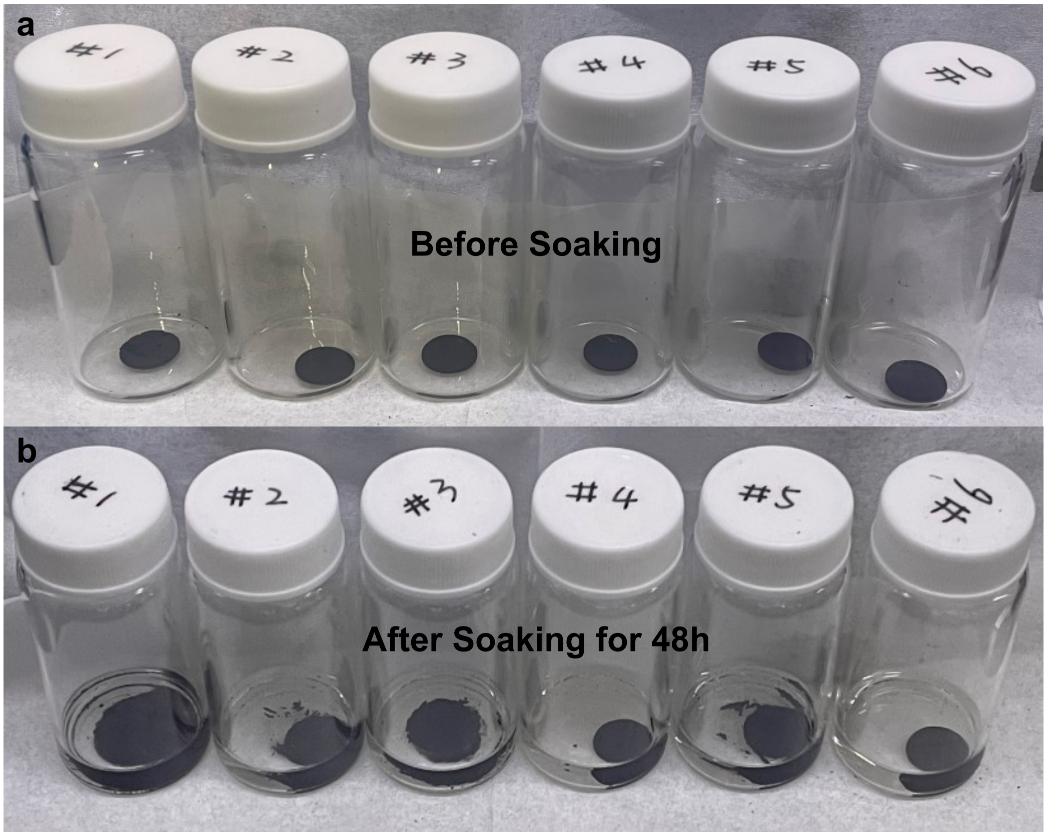

Supplementary Fig. 9. Composite electrodes fabricated using various liquids. a, Composite electrodes after densification process. **b,** The swelling phenomenon of composite electrodes soaking in the liquid electrolyte for 48 hrs. #1: without solvent; #2: with DMF; #3: with acetone; #4: with acetone and DMF; #5: with acetone and IL; #6: with acetone, DMF and IL

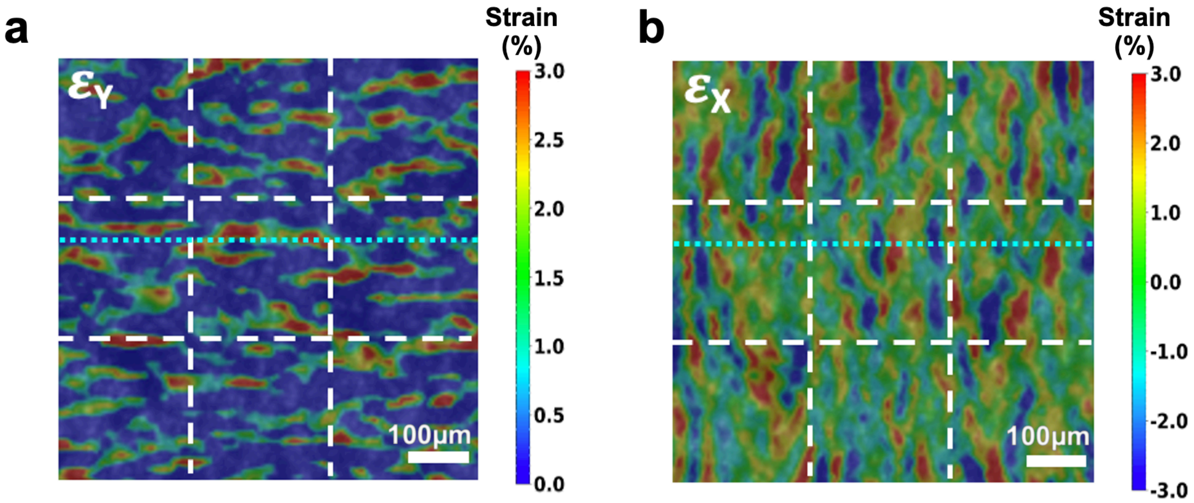

Supplementary Fig. 10. Mechanical characterizations of densified NMC811-PVDF-HFP composite. a, b, Real-time full-field Y-directional (**a**) and X-directional (**b**) strain mapping via Digital Image Correlation (DIC) analysis at a global strain of 0.62% under a uniaxial tensile test (stretching along the Y direction).

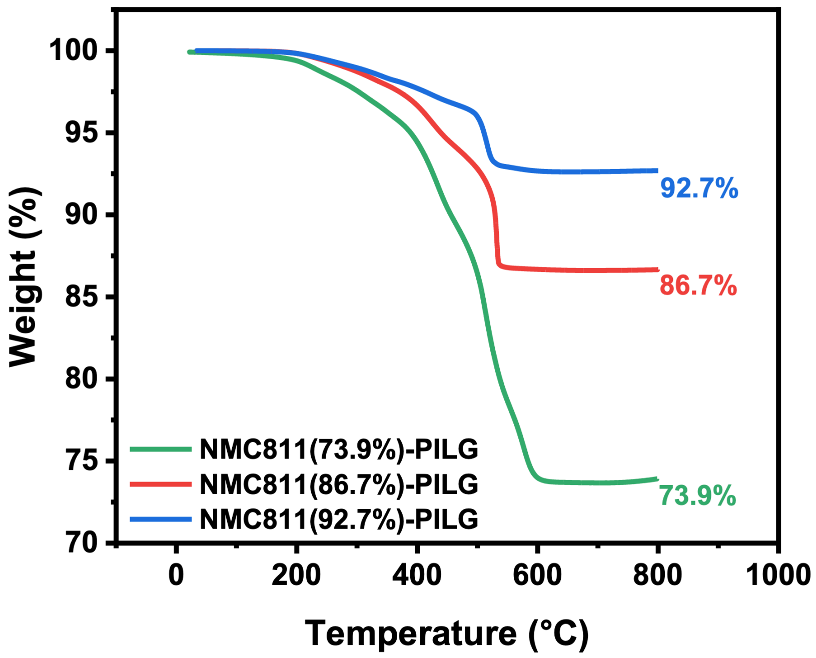

Supplementary Fig. 11. The TGA curves of the NMC811-PILG composites with varying active material contents.

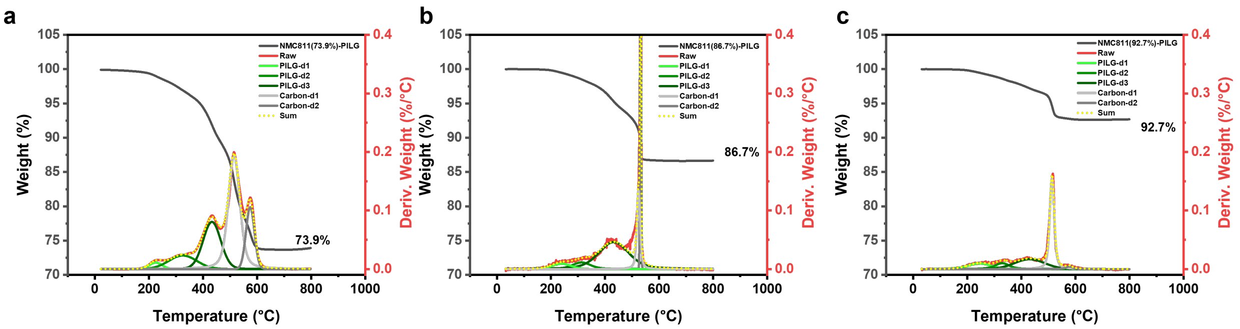

Supplementary Fig. 12. The weight loss curves (left Y-axis) and deconvoluted derivative weight curves (right Y-axis) as a function of temperature for NMC811-PILG composites with different active material contents: 73.9% (**a**), 86.7% (**b**), and 92.7% (**c**).

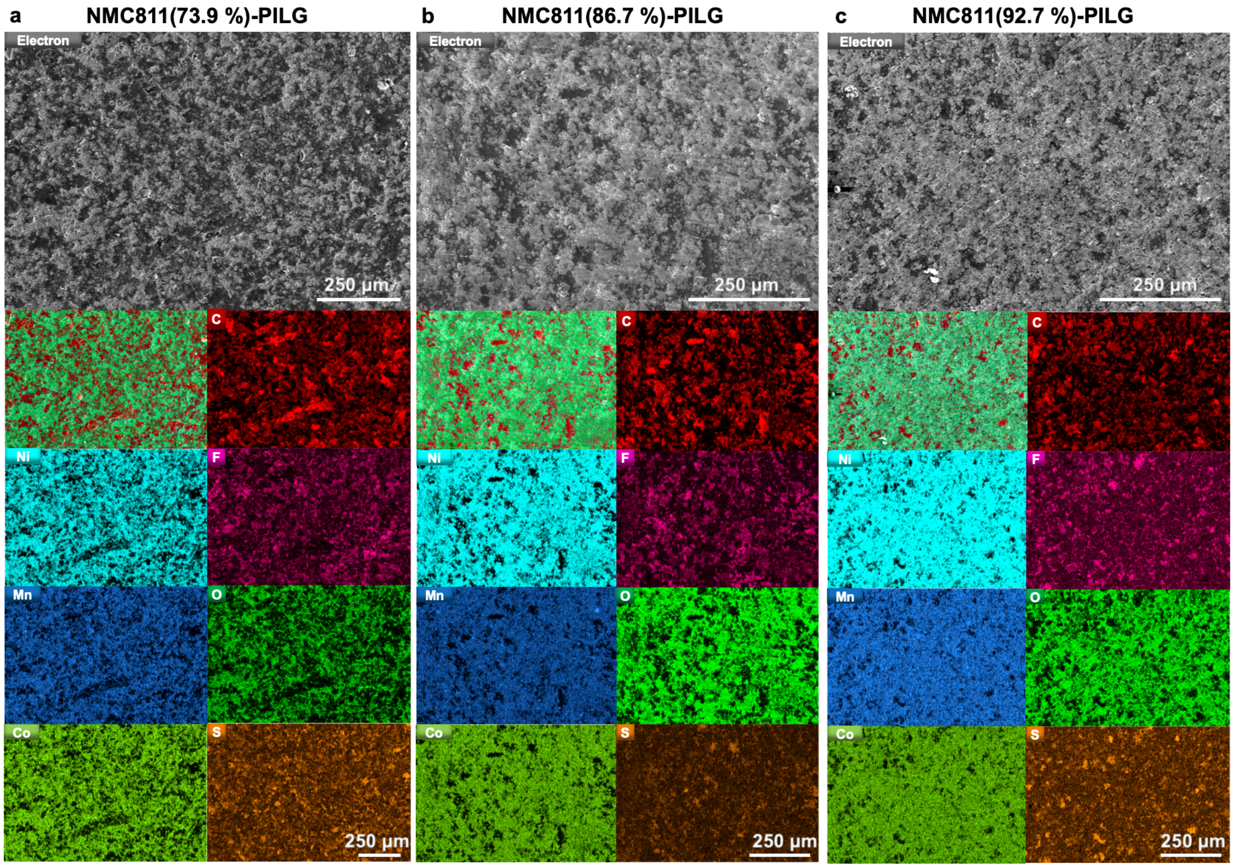

Supplementary Fig. 13. Large-area top-view SEM images and corresponding EDS elemental mapping of all composite constituents acquired on the fractured surface. a, NMC811(73.9%)-PILG composite electrode. **b,** NMC811(86.7%)-PILG composite electrode. **c,** NMC811(92.7%)-PILG composite electrode.

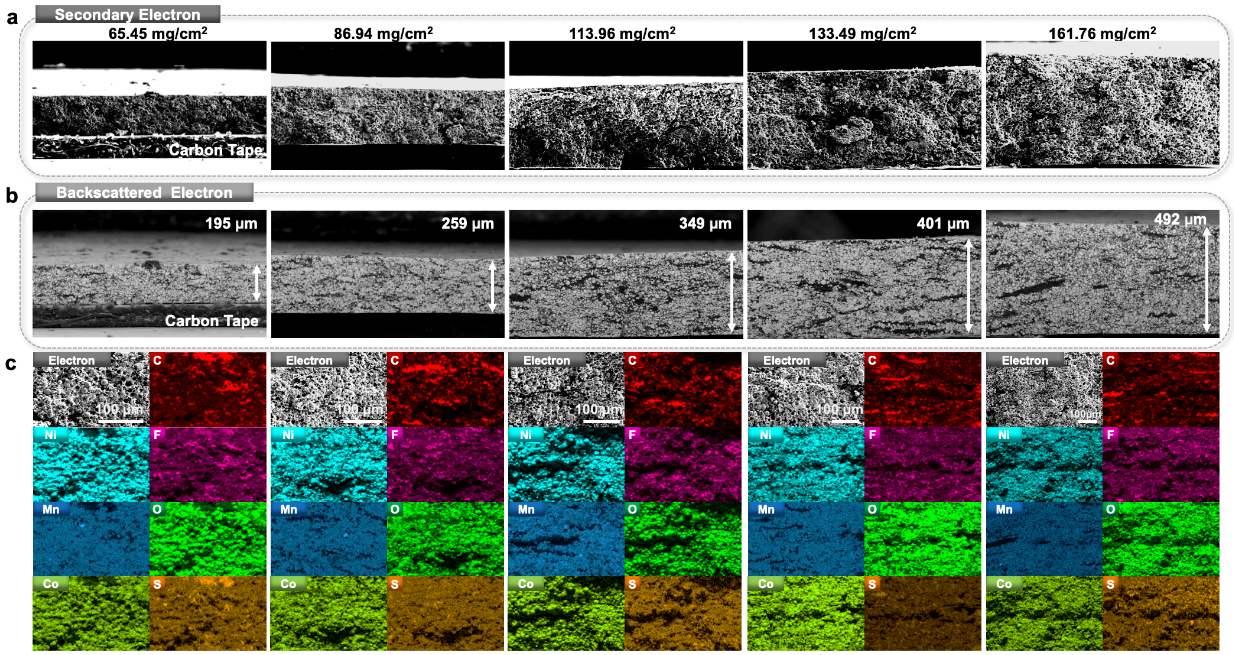

Supplementary Fig. 14. Cross-sectional SEM images and corresponding EDS elemental maps of NMC811 (86.7 wt%)–PILG composites with varying mass loadings (65 mg cm^{-2} to 162 mg cm^{-2}). Secondary electron (**a**) and backscattered electron (**b**) images of fractured cross-sections were acquired at the same magnification after pellet breakage, while EDS elemental maps for C, Ni, F, Mn, O, Co, and S (**c**) were obtained at optimized magnifications for each sample.

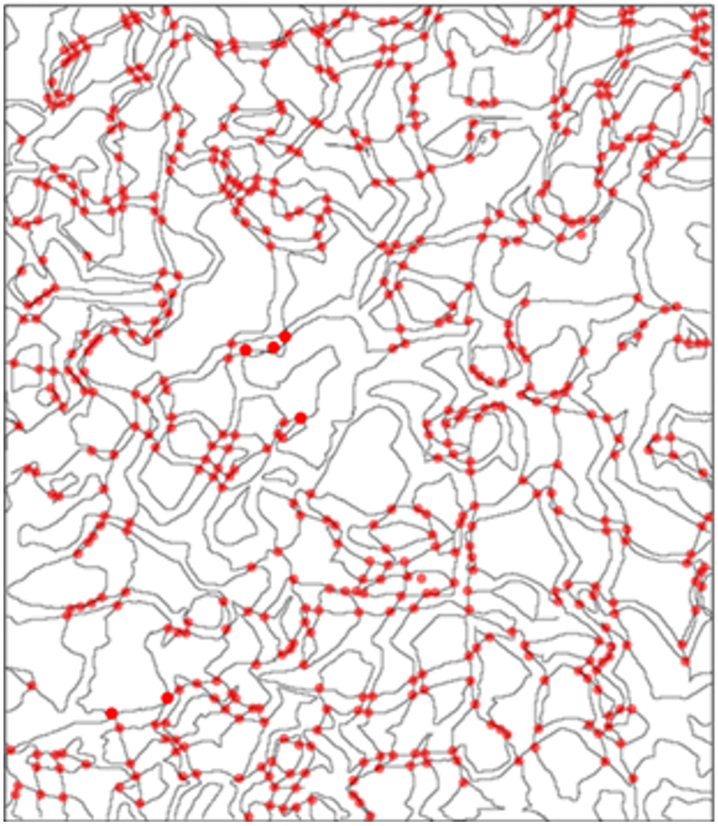

Supplementary Fig. 15. The common 0% strained sites identified by overlaying static interface profiles from both x- and y-strain mappings in the densified NMC811-PILG composite electrode at 25% SOC.

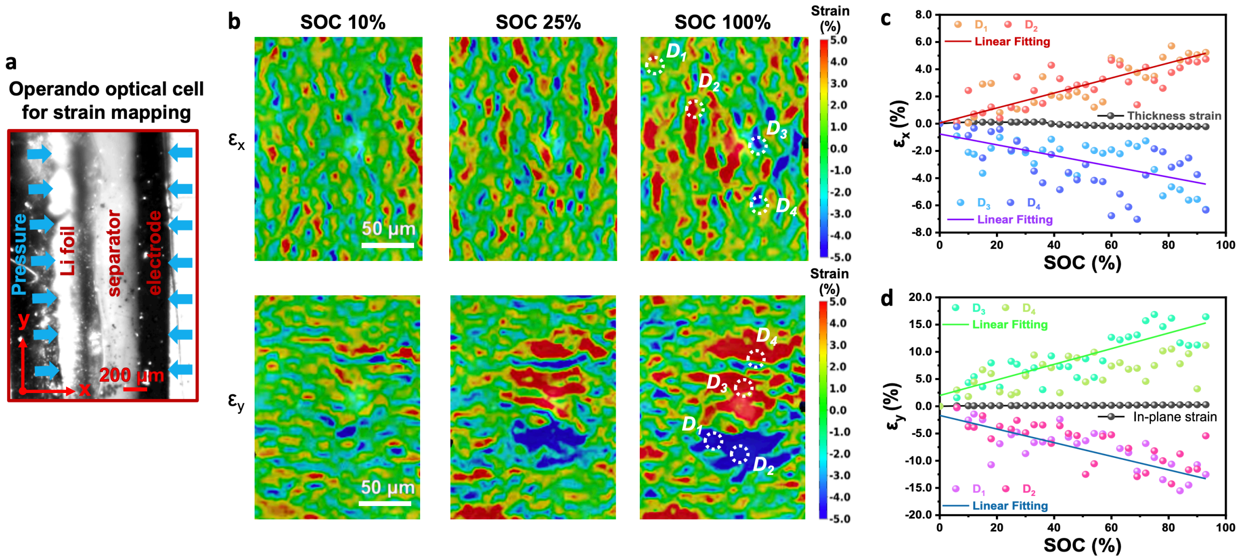

Supplementary Fig. 16. Operando DIC characterizations of electrochemically induced straining. a, Operando optical cell setup for full-field dynamic strain mapping of composite electrodes (300 μm thick) under an applied external force. A thick filter paper was used as the separator. **b**, Full-field x- and y-strain mappings obtained through DIC analysis at various SOCs (0-100%) during the delithiation process at a current of 1.0 mA cm^{-2} under a voltage window of 2.7 – 4.3 V vs. Li│Li^{+}. **c,d**, Electrochemically induced strain measurements of the total electrode thickness along the x-direction, in-plane strain along the y-direction, and representative strain domains at SOCs ranging from 0% to 100%.

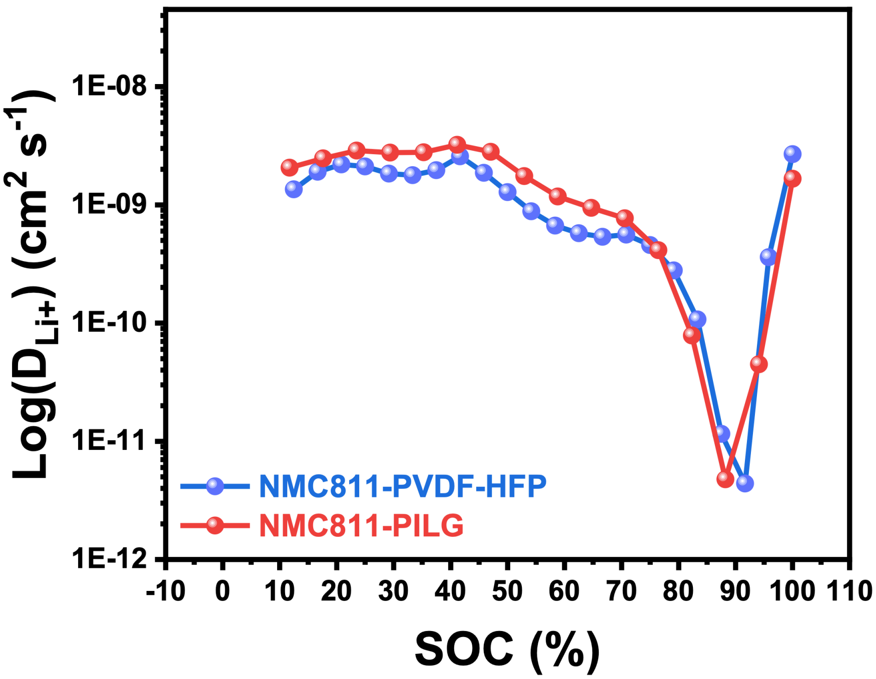

Supplementary Fig. 17. Comparison of GITT results between Li ||NMC811-PILG and Li ||NMC811-PVDF half cells.

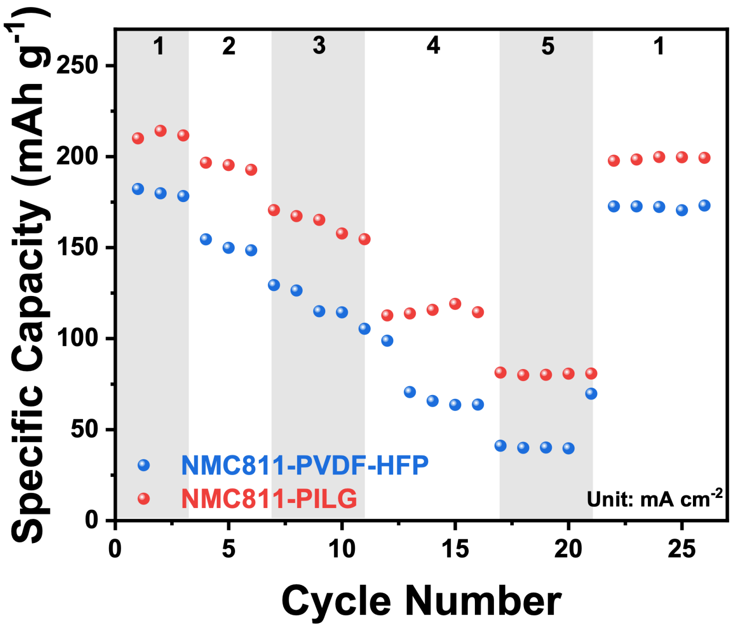

Supplementary Fig. 18. Comparison of the rate performance between NMC811-PILG (with IL) and NMC811-PVDF-HFP (without IL) positive electrodes tested under a voltage window of 2.7 – 4.3 V vs. Li|Li^+.

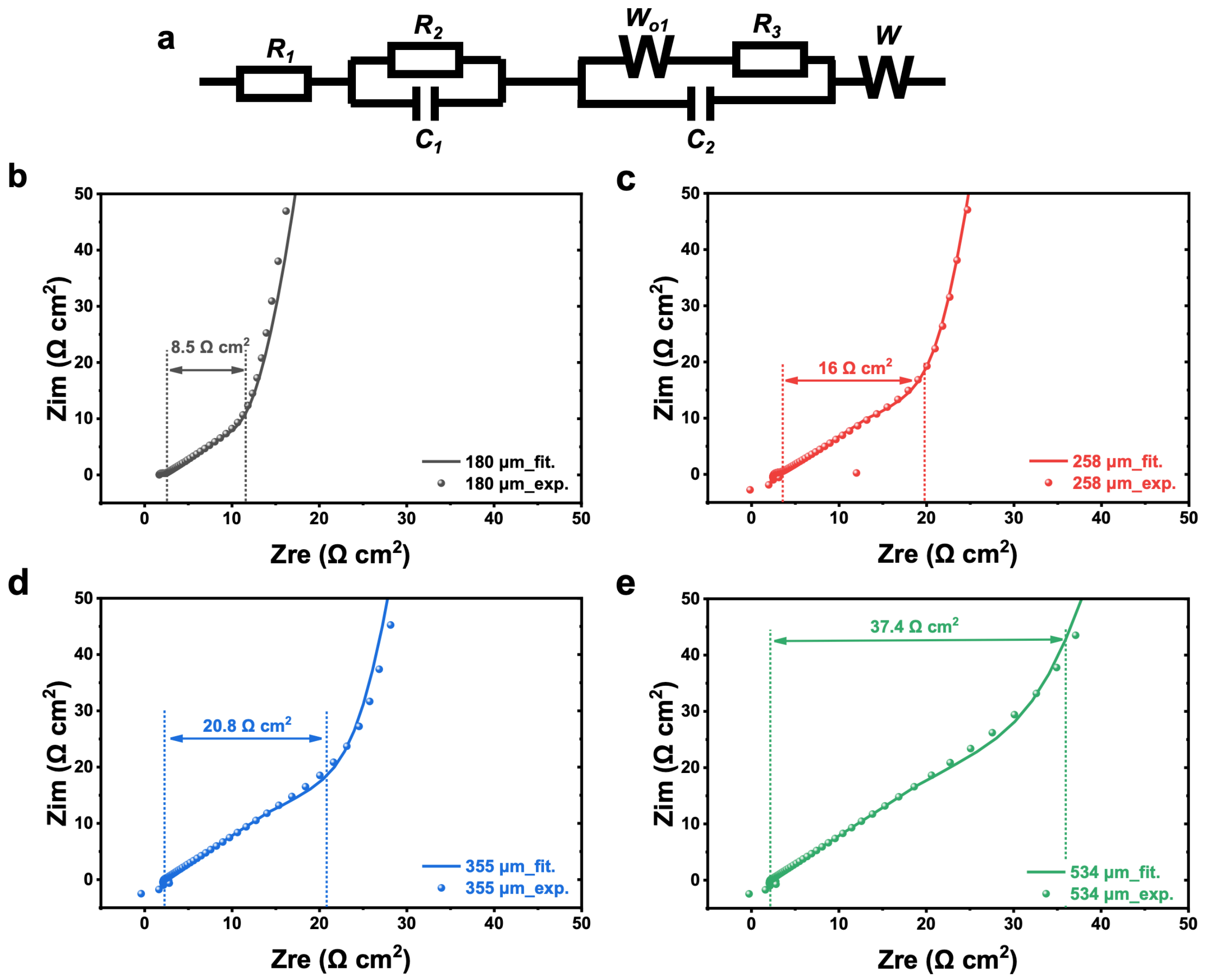

Supplementary Fig. 19. The equivalent circuit (**a**) and Nyquist plots of the symmetric cell (NMC811-PILG||NMC811-PILG) using different electrode thicknesses: **b**, 180 μm; **c**, 258 μm; **d**, 355 μm; and **e**, 534 μm.

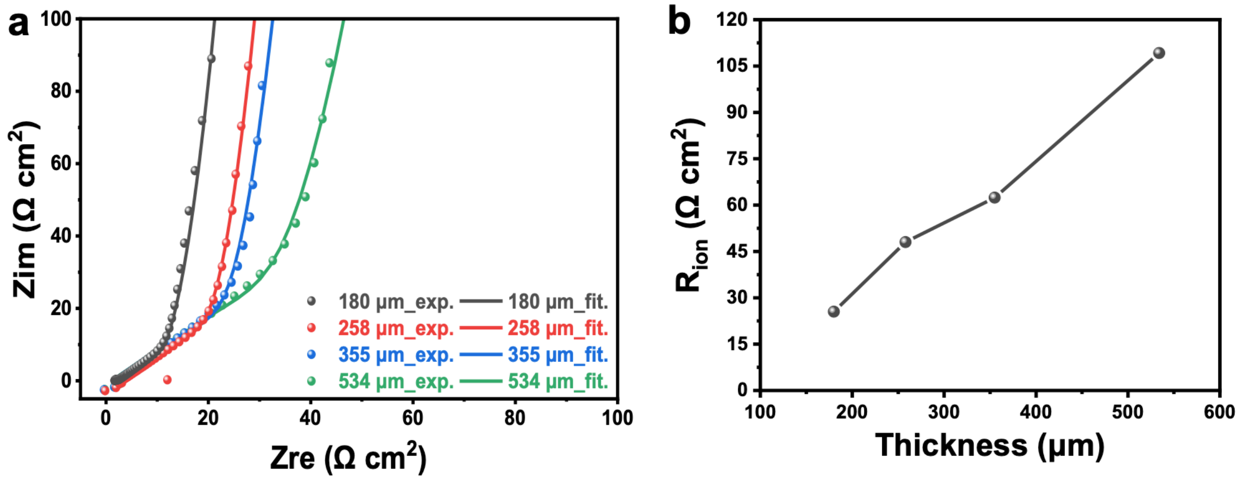

Supplementary Fig. 20. The charge transport kinetics of the electrodes at different thicknesses after the optimized synthetic grain boundary phase. a, the Nyquists plots of NMC811-PILG positive electrodes at different thicknesses; **b**, the relationship between diffusion resistance and the electrode thickness.

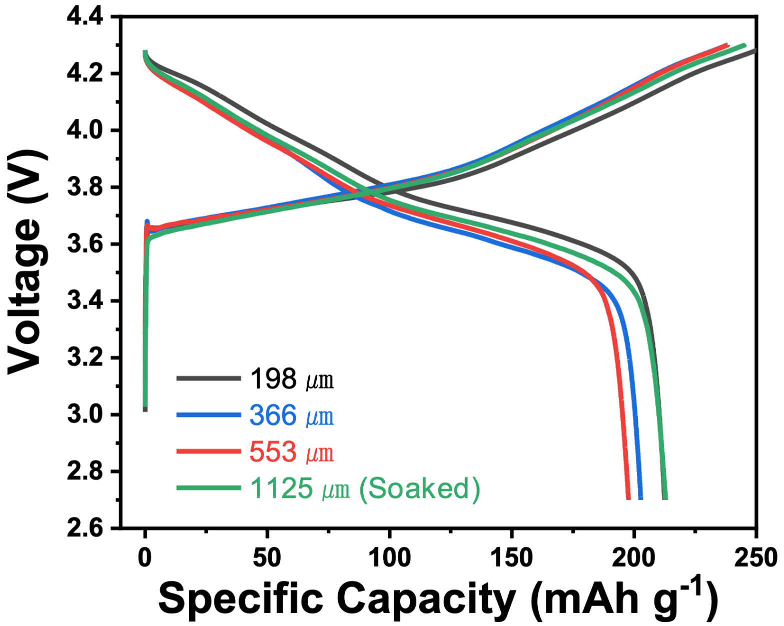

Supplementary Fig. 21. The charge/discharge curve of the positive electrodes with various thicknesses of 198, 366, 553, and 1125 µm at a current density of 1 mA cm^{-2} under a voltage window of 2.7 – 4.3 V vs. Li│Li^{+}.

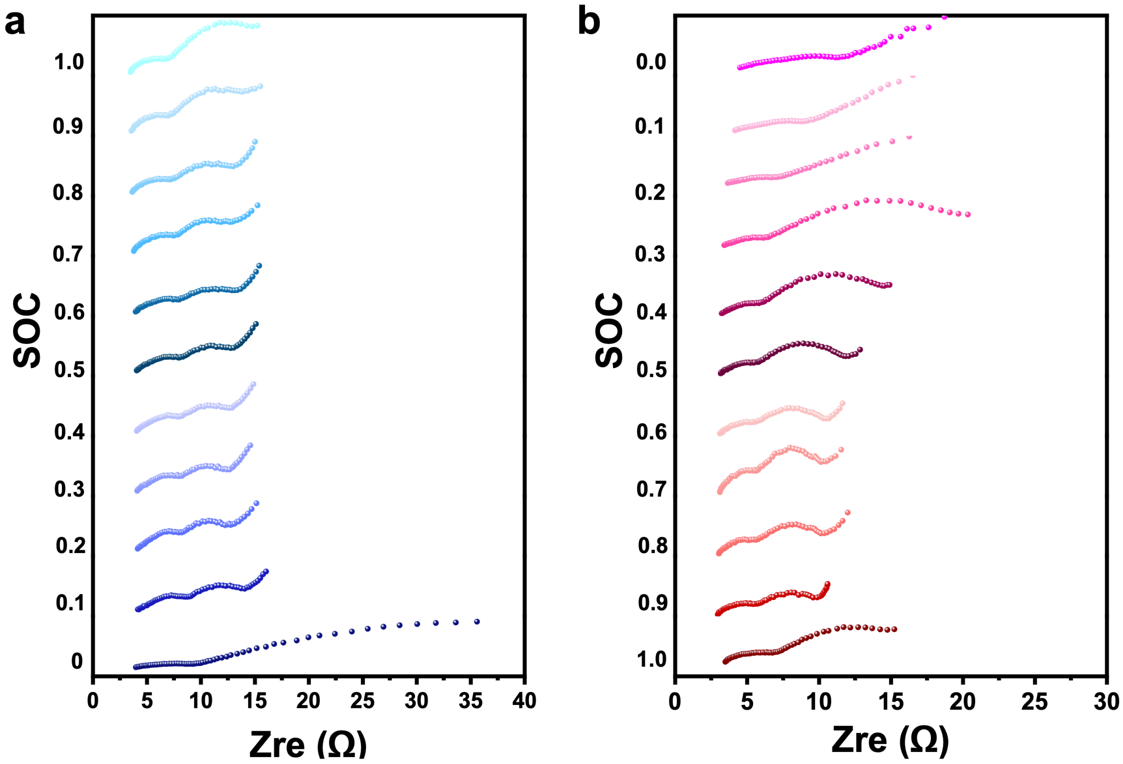

Supplementary Fig. 22. The in-situ EIS during the initial charge (**a**) and discharge (**b**) process of the NMC811-PILG thick positive electrode at an areal current density of 1 mA cm^{-2} under a voltage window of 2.7 – 4.3 V vs. Li | Li^{+}.

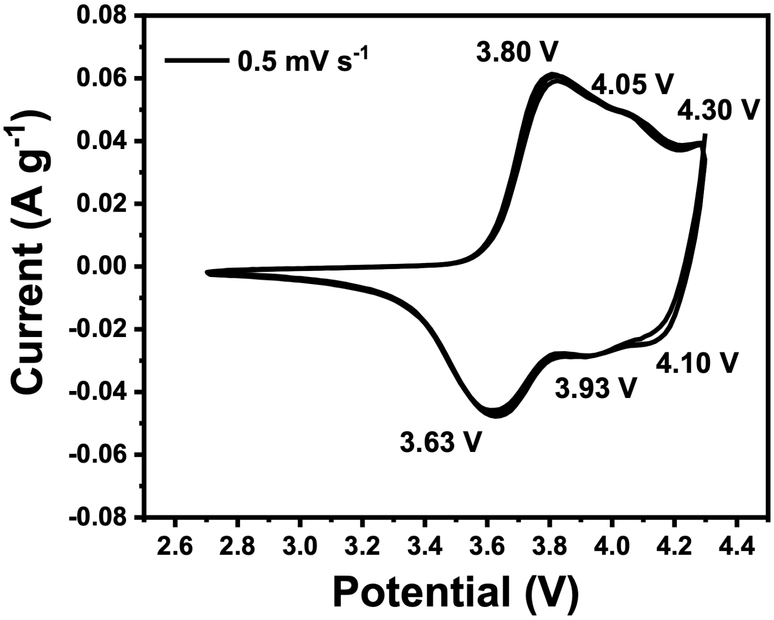

Supplementary Fig. 23. The cyclic voltammetry of the fresh NMC811-PILG positive electrode with a thickness of 204 μm for the first two cycles under a voltage window of 2.7 – 4.3 V vs. Li | Li^+.

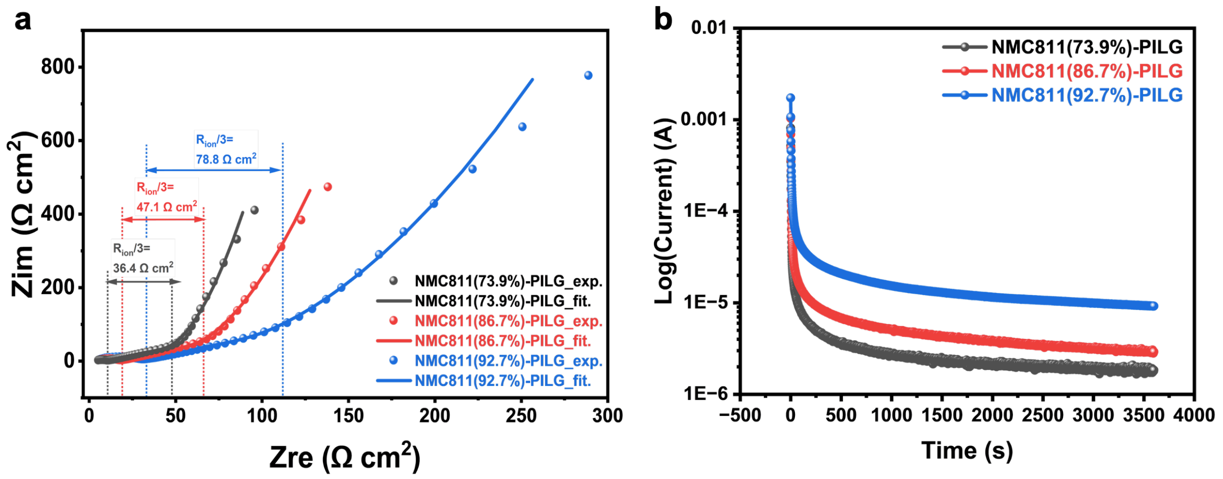

Supplementary Fig. 24. Nyquist plots obtained from potentiostatic EIS of a symmetric cell using two identical electrodes (NMC811-PILG||NMC811-PILG) at 0% SOC (**a**) and DC polarization curves using electron non-blocking electrodes (SUS||NMC811-PILG||SUS) (**b**), with varying active material contents (73.9%, 86.7%, and 92.7%).

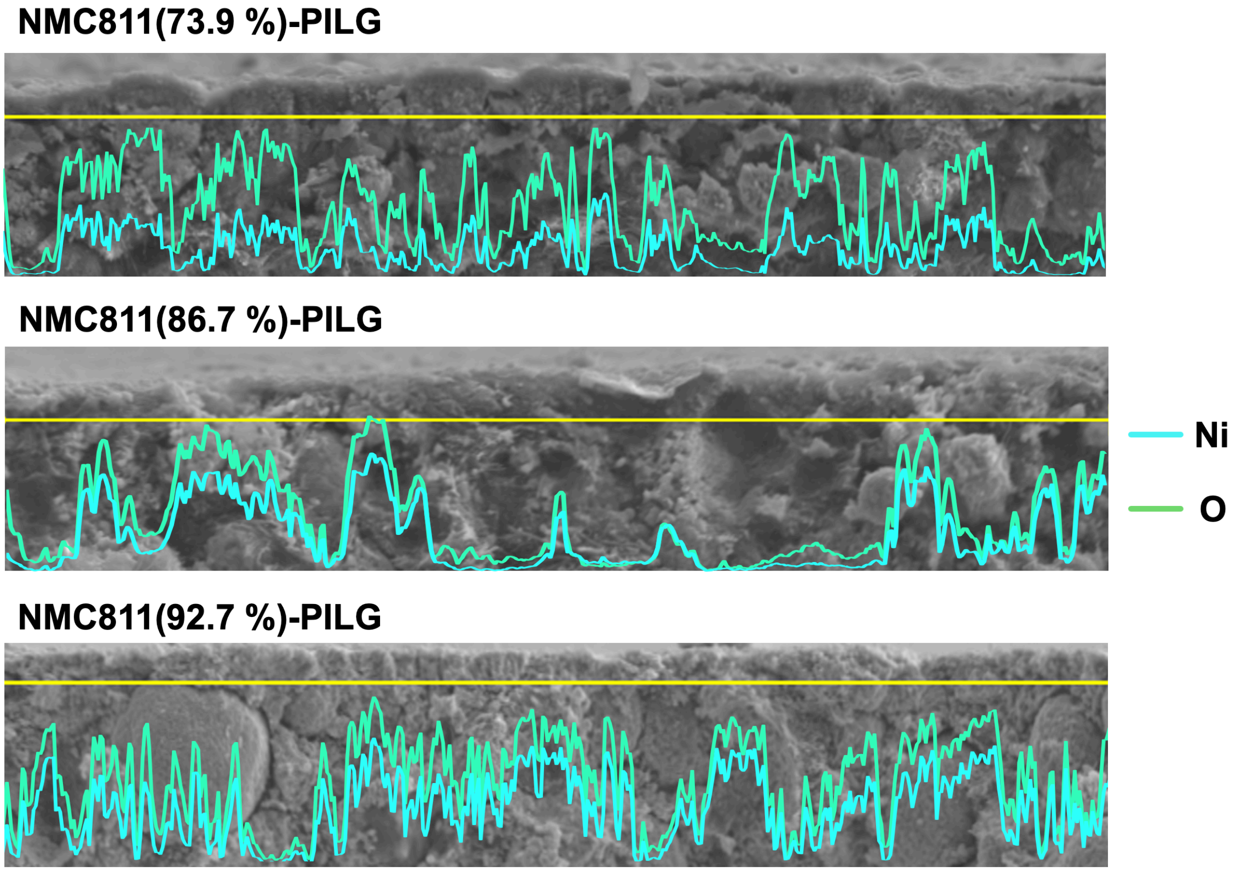

Supplementary Fig. 25. EDS line scan profile of the cross-sectional surface of the cycled sample (>200 cycles at 1 mA cm^{-2}) after vertically cutting, highlighting the distribution of Ni and O elements.

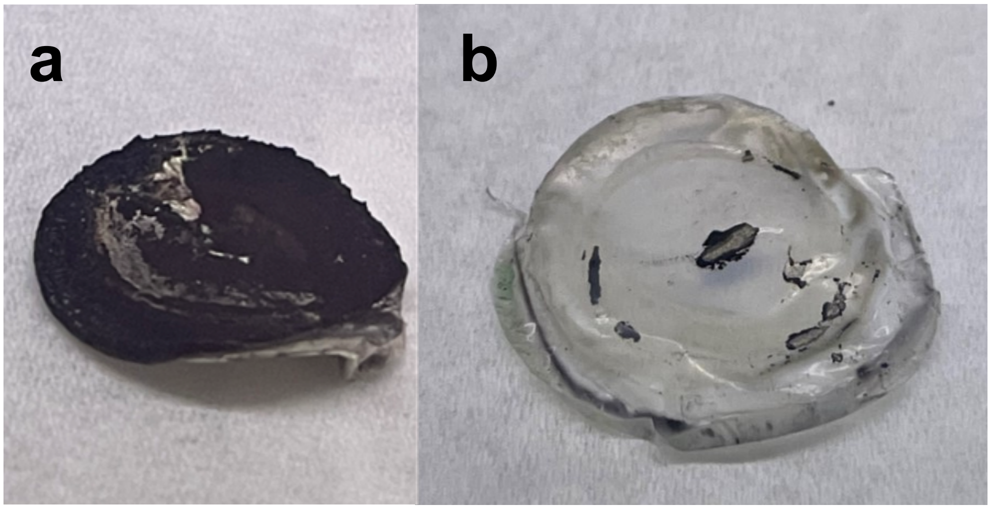

Supplementary Fig. 26. The digital images of the disassembled Li metal (**a**) and separator (**b**) after cycling.

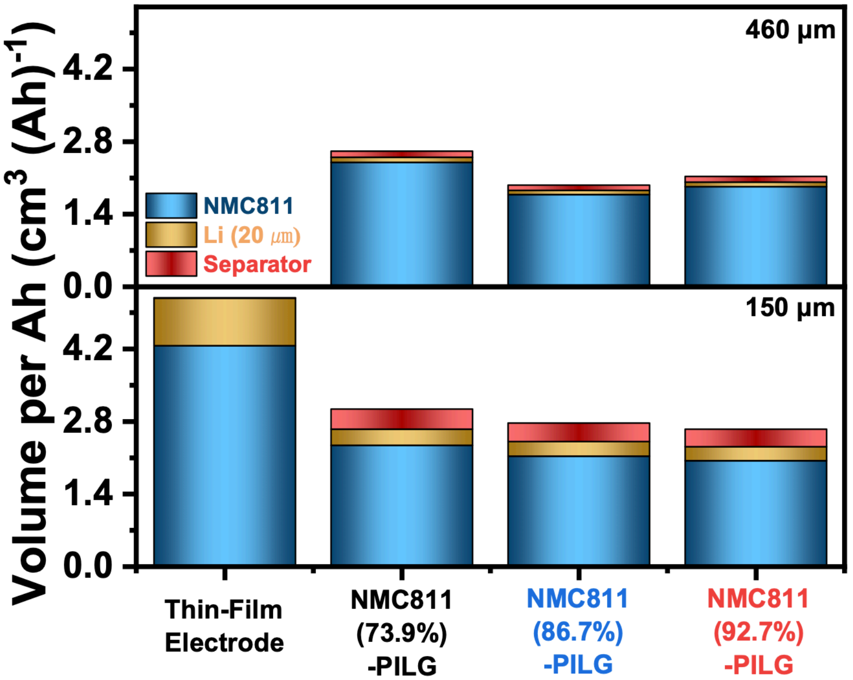

Supplementary Fig. 27. Comparison of the volume per ampere-hour of various active and passive components between slurry-coated thin-film and our NMC811-PILG positive electrodes with different active material loadings (excluding lead and packaging materials).

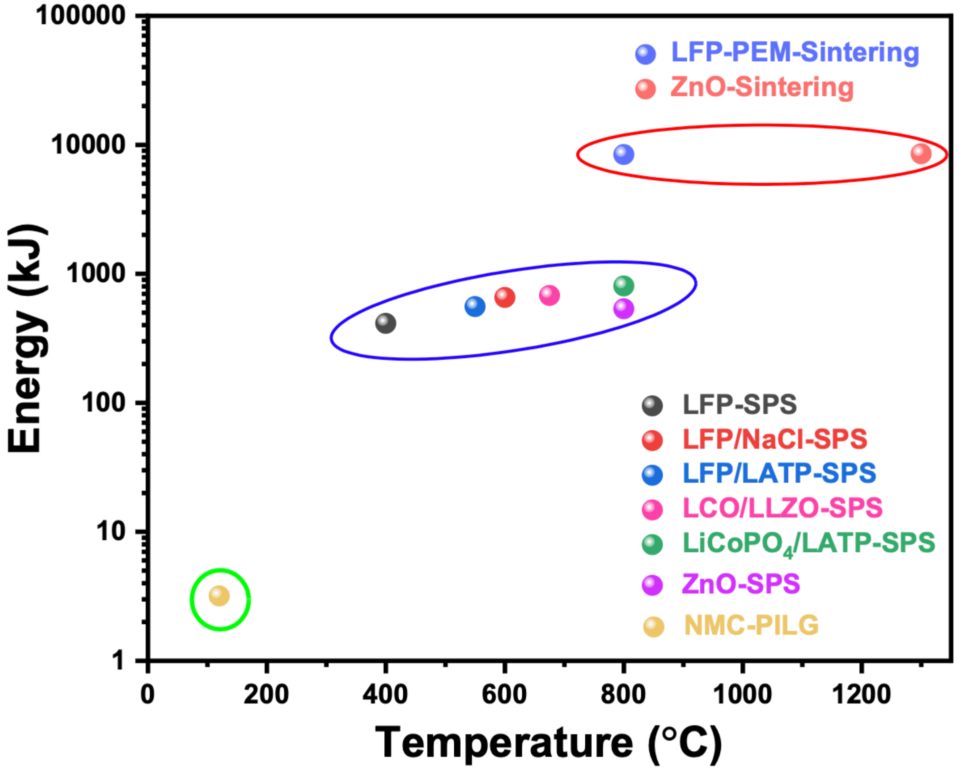

Supplementary Fig. 28. Comparison of energy consumption and processing temperature among various sintering methods (assuming ideal insulating systems) [9-15].

Supplementary Tables

Supplementary Table 1. Precursor weight ratios according to active material content prior to the densification process.

Unit: wt%

Samples	NMC811	CNF	Graphene	PVDF-HFP	EMIMTFSI	LiTFSI
**NMC811(73.9 %)-PILG**	73.0	6.0	3.0	5.0	10.5	2.5
**NMC811(86.7 %)-PILG**	82.0	3.1	2.0	3.6	7.8	1.5
**NMC811(92.7 %)-PILG**	90.0	2.2	1.1	1.9	4.0	0.8

Supplementary Table 2. The decomposition temperature (T_d) and corresponding contents from TGA.

Samples		PILG-d1	PILG-d2	PILG-d3	Carbon-d1	Carbon-d2
**NMC811(73.9 %)-PILG**	T_d (°C)	173-283	198-457	341-529	430-606	526-619
	Weight loss (wt%)	1.50	3.55	9.71	6.25	5.09
**NMC811(86.7 %)-PILG**	T_d (°C)	162-310	270-395	305-572	521-547	496-555
	Weight loss (wt%)	0.60	1.35	5.24	3.63	2.18
**NMC811(92.7 %)-PILG**	T_d (°C)	161-341	267-390	308-546	470-549	557-604
	Weight loss (wt%)	0.60	0.80	3.20	2.00	0.70

Supplementary Table 3. Expected composition ratios of each component are calculated based on the deconvolution results of the derivative weight curves from TGA analysis.

Unit: wt%

Samples	NMC811	CNF	Graphene	PVDF-HFP	EMIMTFSI	LiTFSI
**NMC811(73.9 %)-PILG**	73.90	7.29	3.80	4.20	8.71	2.10
**NMC811(86.7 %)-PILG**	86.70	3.52	2.27	2.20	4.46	0.85
**NMC811(92.7 %)-PILG**	92.70	2.50	1.10	1.01	2.18	0.51

Notes: Ion-conductive phase consists of PVDF-HFP, EMIMTFSI, and LiTFSI; electron-conductive phase consists of CNF and graphene.

Supplementary Table 4. Expected molarities of each component per 1g of the NMC811-PILG positive electrode, calculated based on TGA analysis for each active material content.

Unit: mmol

Samples	NMC811 ($LiNi_{0.8}Mn_{0.1}Co_{0.1}O_2$)	CNF (C)	Graphene (C)	PVDF-HFP ($(CH_2CF_2)_x[CF_2CF(CF_3)]_y$)	EMIMTFSI ($C_8H_{11}F_6N_3O_4S_2$)	LiTFSI ($C_2F_6LiNO_4S_2$)
NMC811 (73.9 %)-PILG	7.60	6.07	3.16	1.05×10^{-4}	2.23×10^{-1}	7.32×10^{-2}
NMC811 (86.7 %)-PILG	8.91	2.93	1.89	5.50×10^{-5}	1.14×10^{-1}	2.96×10^{-2}
NMC811 (92.7 %)-PILG	9.53	2.08	0.92	2.53×10^{-6}	5.57×10^{-2}	1.78×10^{-2}

Supplementary Table 5. Volume ratios of each component calculated from TGA results and from voxel-based analysis of FIB-SEM 3D reconstruction image.

Unit: vol%

**Samples**	**TGA**		**FIB**	
	Components	Calculated volume ratio	Components	Calculated volume ratio
**NMC811(86.7 %)-PILG**	NMC811	69.78	NMC811	65.28
	CNF	6.90	PILG	26.30
	Graphene	4.45		
	PVDF-HFP	4.92		
	EMIMTFSI	11.46		
	LiTFSI	2.50		
	Pores	N/A	Pores	8.42

* The FIB-SEM experiment was conducted on a sample with thickness of 362μm and a relative density of 87.4%.

Supplementary Table 6. Lithium-ion diffusion resistance R_{ion} of thick positive electrodes at different thicknesses.

Electrode	Thickness (μm)	$R_{ion}/3$ (Ohm cm^2)	R_{ion} (Ohm cm^2)	Fitting error (%)
NMC(73.9%)-PILG	180	8.5	25.5	6.1
NMC(73.9%)-PILG	258	16.0	48.0	16.0
NMC(73.9%)-PILG	355	20.8	62.4	14.8
NMC(73.9%)-PILG	387	36.4	109.2	1.4
NMC(73.9%)-PILG	534	37.4	112.2	17.3
NMC(86.7%)-PILG	373	47.1	141.3	1.9
NMC(92.7%)-PILG	335	78.8	236.4	3.7
NMC-PVDF-HFP	175	20.5	61.5	2.2

Supplementary Table 7 The electrochemical performance of prior thick positive electrodes.

Cathode	Thickness (μm)	Porosity	Mass Loading (mg cm^{-2})	Current Density (mA cm^{-2}/C)	Areal Capacity (mAh cm^{-2})	Vol. Capacity (mAh cm^{-3})	Grav. Capacity (mAh g^{-1})	Ref.
Vertically lamellar NMC111	1200	0.40	240	3.3/0.1	32.0	266.7	137.0	7
NMC811/ CNTs	800	0.55	155	1.5/0.05	29.0	362.5	187.1	16
Ultrathick bulk LCO	1500	0.45	187.5	1.4/0.05	24.5	163.3	130.7	17
Thick-porous LFP	1000	0.40	150	1.3/0.05	21.0	210.0	140.0	11
LFP/CNFs	947	0.38	126	1.1/0.05	21.4	226.0	169.8	18
FAT LFP	1000	0.40	128	1.1/0.05	20.0	200.0	156.3	19
UCFR LFP	1350	0.63	108	0.9/0.05	16.4	121.5	151.9	20
Bi/trilayer LFP-PESF	1200	0.71	100	0.9/0.05	15.6	130.0	156.0	21
UCT-LFP	1300	0.40	100	1.7/0.1	15.1	116.2	151.0	22
Low-tortuosity LCO	440	0.66	100.5	0.7/0.05	14.0	318.2	139.3	23
Ice-templated LFP	900	0.36	80	0.7/0.05	13.0	144.4	162.5	24
NS-NMC111	1710	0.38	320	5.1/0.1	45.4	265.5	141.9	25
TIPS-NMC111	2000	0.82	136	1.1/0.05	20.4	102.0	150.0	26
NMC	360	0.12-0.14	62	1.0/0.06	15.9	434	203	This Work
	360		62	2.0/0.13	14.3	391	180	
	530		91	1.0/0.04	23.1	420	195	
	1125		187	1.0/0.02	49.7	440	213	

Supplementary Table 8. The mass and volume configuration of the battery half-cell with the single-side coated thin film positive electrode (Li||NMC811).

Components	Materials	Mass weight (g $(Ah)^{-1}$)	Layer thickness (μm)	Volume (cm^3 $(Ah)^{-1}$)
Positive electrode	NMC811	5	100	4.2606
	Carbon black	0.59		
	PVDF	0.29		
	Al	1.54		
Electrolyte	LiPF6/EC-DMC	1.19	NA	NA
	PP/PE	0.12	25	0.0061
Negative electrode	Li	0.2	20	0.9182

* The single-side coated thin-film electrode has a composition of NMC811:C:PVDF = 0.85:0.1:0.05.

Supplementary Table 9. The mass and volume configuration of the battery half-cell with the thick positive electrode (Li||NMC811-PILG).

Samples	Components	Materials	Mass weight (g (Ah)$^{-1}$)	Layer thickness (μm)	Volume (cm^3 (Ah)$^{-1}$)
**NMC811(73.9%)-PILG**	Positive electrode	NMC811	4.59	150	2.34
		Carbon	0.69		
		PILG	0.93		
		Al	0		
	Electrolyte	$LiPF_6$/EC-DMC	0.47	NA	NA
		PP/PE	0.19	25	0.39
	Negative electrode	Li	0.17	20	0.31
**NMC811(86.7%)-PILG**	Positive electrode	NMC811	5.23	150	2.13
		Carbon	0.35		
		PILG	0.45		
		Al	0		
	Electrolyte	$LiPF_6$/EC-DMC	0.43	NA	NA
		PP/PE	0.17	25	0.36
	Negative electrode	Li	0.15	20	0.28
**NMC811(92.7%)-PILG**	Positive electrode	NMC811	5.94	150	2.04
		Carbon	0.23		
		PILG	0.24		
		Al	0		

Electrolyte	$LiPF_6$/EC-DMC	0.41	NA	NA
	PP/PE	0.16	25	0.34
Negative electrode	Li	0.15	20	0.27

Supplementary Table 10. The mass and volume configuration of the battery half-cell using ultrathick electrodes.

Samples	Components	Materials	Mass weight (g (Ah)$^{-1}$)	Layer thickness (μm)	Volume (cm^3 (Ah)$^{-1}$)
**NMC811(73.9%)-PILG**	Positive electrode	NMC811	4.44	488	2.40
		Carbon	0.66		
		PILG	0.90		
		Al	0		
	Electrolyte	$LiPF_6$/EC-DMC	0.15	NA	NA
		PP/PE	0.06	25	0.12
	Negative electrode	Li	0.05	20	0.098
**NMC811(86.7%)-PILG**	Positive electrode	NMC811	4.91	461	1.78
		Carbon	0.33		
		PILG	0.43		
		Al	0		
	Electrolyte	$LiPF_6$/EC-DMC	0.12	NA	NA
		PP/PE	0.05	25	0.1
	Negative electrode	Li	0.04	20	0.08
**NMC811(92.7%)-PILG**	Positive electrode	NMC811	5.94	442	1.93
		Carbon	0.03		
		PILG	0.24		
		Al	0		

Electrolyte	$LiPF_6$/EC-DMC	0.13	NA	NA
	PP/PE	0.05	25	0.11
Negative electrode	Li	0.05	20	0.09

Supplementary References

1 McGrath, L. M., Jones, J., Carey, E. & Rohan, J. F. Ionic Liquid Based Polymer Gel Electrolytes for Use with Germanium Thin Film Anodes in Lithium Ion Batteries. *ChemistryOpen* **8**, 1429-1436 (2019). https://doi.org/https://doi.org/10.1002/open.201900313

2 Tseng, Y.-C. *et al.* Polymer electrolytes based on Poly(VdF-co-HFP)/ionic liquid/carbonate membranes for high-performance lithium-ion batteries. *Polymer* **173**, 110-118 (2019). https://doi.org/https://doi.org/10.1016/j.polymer.2019.04.008

3 Mo, Y., Wan, Y., Chau, A. & Huang, F. Graphene/Ionic Liquid Composite Films and Ion Exchange. *Scientific Reports* **4**, 5466 (2014). https://doi.org/10.1038/srep05466

4 Aldroubi, S., Brun, N., Bou Malham, I. & Mehdi, A. When graphene meets ionic liquids: a good match for the design of functional materials. *Nanoscale* **13**, 2750-2779 (2021). https://doi.org/10.1039/D0NR06871C

5 Farivar, F. *et al.* Unlocking thermogravimetric analysis (TGA) in the fight against "Fake graphene" materials. *Carbon* **179**, 505-513 (2021). https://doi.org/https://doi.org/10.1016/j.carbon.2021.04.064

6 Ogihara, N., Itou, Y., Sasaki, T. & Takeuchi, Y. Impedance Spectroscopy Characterization of Porous Electrodes under Different Electrode Thickness Using a Symmetric Cell for High-Performance Lithium-Ion Batteries. *The Journal of Physical Chemistry C* **119**, 4612-4619 (2015). https://doi.org/10.1021/jp512564f

7 Ju, Z. *et al.* Densified vertically lamellar electrode architectures for compact energy storage. *Proceedings of the National Academy of Sciences* **120**, e2308009120 (2023). https://doi.org/10.1073/pnas.2308009120

8 Sohrabi Baba Heidary, D., Lanagan, M. & Randall, C. A. Contrasting energy efficiency in various ceramic sintering processes. *Journal of the European Ceramic Society* **38**, 1018-1029 (2018). https://doi.org/https://doi.org/10.1016/j.jeurceramsoc.2017.10.015

9 Nie, B. *et al.* Alignment does matter: Design thick electrodes to improve the comprehensive lithium storage performance. *Carbon* **206**, 105-113 (2023). https://doi.org/https://doi.org/10.1016/j.carbon.2023.02.015

10 Tong, H., Liu, J., Qiao, Y., Amardeep, A. & Song, X. Microstructure and electrochemistry performance of the composite electrode prepared by spark plasma sintering. *Journal of the European Ceramic Society* **43**, 419-427 (2023). https://doi.org/https://doi.org/10.1016/j.jeurceramsoc.2022.09.058

11 Elango, R., Demortière, A., De Andrade, V., Morcrette, M. & Seznec, V. Thick Binder-Free Electrodes for Li–Ion Battery Fabricated Using Templating Approach and Spark Plasma Sintering Reveals High Areal Capacity. *Advanced Energy Materials* **8**, 1703031 (2018). https://doi.org/https://doi.org/10.1002/aenm.201703031

12 Laptev, A. M., Zheng, H., Bram, M., Finsterbusch, M. & Guillon, O. High-pressure field assisted sintering of half-cell for all-solid-state battery. *Materials Letters* **247**, 155-158 (2019). https://doi.org/https://doi.org/10.1016/j.matlet.2019.03.109

13 Okumura, T., Takeuchi, T. & Kobayashi, H. Application of LiCoPO4 Positive Electrode Material in All-Solid-State Lithium-Ion Battery. *Electrochemistry* **82**, 906-908 (2014). https://doi.org/10.5796/electrochemistry.82.906

14 Sotomayor, M. E. *et al.* Ultra-thick battery electrodes for high gravimetric and volumetric energy density Li-ion batteries. *Journal of Power Sources* **437**, 226923 (2019). https://doi.org/https://doi.org/10.1016/j.jpowsour.2019.226923

15 Zhao, X. *et al.* Comparative study on energy efficiency and densification of ZnO ceramics using various sintering processes. *Journal of Materials Science: Materials in Electronics* **34**, 1798 (2023). https://doi.org/10.1007/s10854-023-11184-1

16 Park, S.-H. *et al.* High areal capacity battery electrodes enabled by segregated nanotube networks. *Nature Energy* **4**, 560-567 (2019). https://doi.org/10.1038/s41560-019-0398-y

17 Lu, L.-L. *et al.* Wood-Inspired High-Performance Ultrathick Bulk Battery Electrodes. *Advanced Materials* **30**, 1706745 (2018). https://doi.org/https://doi.org/10.1002/adma.201706745

18 Wu, J. *et al.* Building Efficient Ion Pathway in Highly Densified Thick Electrodes with High Gravimetric and Volumetric Energy Densities. *Nano Letters* **21**, 9339-9346 (2021). https://doi.org/10.1021/acs.nanolett.1c03724

19 Shi, B. *et al.* Low Tortuous, Highly Conductive, and High-Areal-Capacity Battery Electrodes Enabled by Through-thickness Aligned Carbon Fiber Framework. *Nano Letters* **20**, 5504-5512 (2020). https://doi.org/10.1021/acs.nanolett.0c02053

20 Li, H. *et al.* Ultrahigh-Capacity and Fire-Resistant LiFePO4-Based Composite Cathodes for Advanced Lithium-Ion Batteries. *Advanced Energy Materials* **9**, 1802930 (2019). https://doi.org/https://doi.org/10.1002/aenm.201802930

21 Wu, J. *et al.* Ultrahigh-Capacity and Scalable Architected Battery Electrodes via Tortuosity Modulation. *ACS Nano* **15**, 19109-19118 (2021). https://doi.org/10.1021/acsnano.1c06491

22 Wang, J. *et al.* High-areal-capacity thick cathode with vertically-aligned micro-channels for advanced lithium ion batteries. *Energy Storage Materials* **39**, 287-293 (2021). https://doi.org/https://doi.org/10.1016/j.ensm.2021.04.030

23 Li, L., Erb, R. M., Wang, J., Wang, J. & Chiang, Y.-M. Fabrication of Low-Tortuosity Ultrahigh-Area-Capacity Battery Electrodes through Magnetic Alignment of Emulsion-Based Slurries. *Advanced Energy Materials* **9**, 1802472 (2019). https://doi.org/https://doi.org/10.1002/aenm.201802472

24 Huang, C., Dontigny, M., Zaghib, K. & Grant, P. S. Low-tortuosity and graded lithium ion battery cathodes by ice templating. *Journal of Materials Chemistry A* **7**, 21421-21431 (2019). https://doi.org/10.1039/C9TA07269A

25 Wu, T. *et al.* Thick electrode with thickness-independent capacity enabled by assembled two-dimensional porous nanosheets. *Energy Storage Materials* **36**, 265-271 (2021). https://doi.org/https://doi.org/10.1016/j.ensm.2020.12.034

26 Lee, J. T., Jo, C. & De Volder, M. Bicontinuous phase separation of lithium-ion battery electrodes for ultrahigh areal loading. *Proceedings of the National Academy of Sciences* **117**, 21155-21161 (2020). https://doi.org/10.1073/pnas.2007250117
